# Supplementary material for: Collaborative intelligence in AI: Evaluating the performance of a council of AIs on the USMLE
Source: PLOS Digit Health. 2025 Oct 9;4(10):e0000787. doi: 10.1371/journal.pdig.0000787 (PMC12510544; doi:10.1371/journal.pdig.0000787)
Supplement: S2 File — (ZIP) [file pdig.0000787.s002.zip › S2 Data Raw data files containing unprocessed question inputs and LLM outputs including transcripts of deliberations/STEP3_Question-80-1_Type-MC-NJ.docx]

**Facilitator** **[Round 1]:** A 9-year-old boy is brought to the office by his parents to establish care after recently moving to the area. The patient has not been evaluated by a physician in 4 years. He has been generally healthy. Medical history is significant for pneumonia at age 3 years. He takes no medications. He is at the 25th percentile for height, weight, and BMI. Vital signs are temperature 37.0°C (98.6°F), pulse 82/min, respirations 20/min, and blood pressure 112/74 mm Hg. Cardiac examination discloses a grade 3/6 systolic murmur audible along the left sternal border at the third and fourth intercostal spaces. Femoral pulses are weak and brachial pulses are strong; there is a radiofemoral delay. Chest x-ray discloses mild cardiomegaly with left ventricular prominence. ECG shows left ventricular hypertrophy. This patient is at greatest risk for which of the following complications? (A) Atrial fibrillation (B) Cor pulmonale (C) Systemic hypertension (D) Tricuspid valve regurgitation

**AI_C:** RESTATING THE QUESTION: This case involves a 9-year-old boy who presents with a health history highlighting a grade 3/6 systolic murmur, weak femoral pulses and strong brachial pulses, radiofemoral delay, noticeable cardiomegaly with left ventricular prominence and ECG demonstrating left ventricular hypertrophy. We're tasked with determining the greatest risk of a potential complication from the provided options: Atrial fibrillation, Cor pulmonale, Systemic hypertension, or Tricuspid valve regurgitation. PLANNING: The assumptions are that the physical examination, the chest X-ray, and the ECG findings are significant for assessing the risk of complications for this child. The noticeable systolic murmur, radiofemoral delay, and evidence of left ventricular hypertrophy can suggest possible aortic coarctation, a narrowing of the large blood vessel branching off the heart. CLINICAL REASONING: Aortic coarctation presents with high pressure before the constriction point (upper body), hence the strong brachial pulses and lower pressure after the obstruction (lower body), hence weak femoral pulses and a radiofemoral delay. This scenario also typically involves left ventricular hypertrophy due to increased ventricular workload from systemic hypertension. Mild cardiomegaly noted could represent the heart's response to chronic pressure overload. Therefore, among the options given, systemic hypertension seems to be the most likely complication as the left ventricle pumps against a higher systemic vascular resistance due to the narrowed aorta. CONCLUDING: Given the clinical data indicative of a potential aortic coarctation given the presence of a grade 3/6 systolic murmur, altered pulse strength (strong brachial and weak femoral), radiofemoral delay, mild cardiomegaly with ventricular prominence, and ECG proof of left ventricular hypertrophy, the primary complication likely to occur is systemic hypertension. RESPONSE: The most likely complication this patient faces is Systemic hypertension (C).

**AI_B:** RESTATING THE QUESTION: The query asks us to find out which complication this 9-year-old boy is likeliest to suffer from, given examination notes mentioning a grade 3/6 systolic murmur, a radiofemoral delay, mild cardiomegaly shown by an x-ray, and left ventricular hypertrophy evidenced by an ECG reading. PLANNING: The significant clinical findings for potential assumptions include: systolic murmur, radiofemoral delay, mild cardiomegaly, and left ventricular hypertrophy. Systolic murmur could imply a cardiac valve issue; radiofemoral delay may suggest coarctation of the aorta; mild cardiomegaly typically points to a heart disorder, and left ventricular hypertrophy is often an outcome of increased afterload or volume overload. Clinical reasoning: The combination of these signs suggests a structural heart abnormality. The presence of a systolic murmur along with the radiofemoral delay points to aortic coarctation, a congenital heart disease marked by a narrowing of the aorta. The left ventricular hypertrophy and mild cardiomegaly could be a compensatory response to this narrowing, as the left ventricle works harder to pump blood against the increased afterload. This might eventually contribute to systemic hypertension. CONCLUDING: Therefore, in the context of an otherwise healthy 9-year-old presenting with these particular clinical findings, it may be hypothesized that the patient's complicated cardiac presentation may be indicative of aortic coarctation, with a high risk of developing systemic hypertension due to the increased effort of the left ventricle to overcome the stenosis. RESPONSE: The patient is at greatest risk for Systemic Hypertension (Option C).

**AI_E:** RESTATING THE QUESTION: The case is about a 9-year-old boy with a history of pneumonia and no recent doctor visits in the last 4 years. He's small for his age and has abnormal cardiac findings -- a systolic heart murmur, weak femoral pulses compared to strong brachial pulses (radiofemoral delay), cardiomegaly with left ventricular prominence on chest x-ray and left ventricular hypertrophy on ECG. With these findings, we're being asked to determine his greatest health risk. The options include atrial fibrillation (irregular rapid heart rate that can lead to stroke, heart failure), Cor pulmonale (right-sided heart failure due to high blood pressure in the lungs), systemic hypertension (high blood pressure affecting the whole body), and tricuspid valve regurgitation (backward flow of blood into the right atrium). PLANNING: The presence of radiofemoral delay, along with the grade 3/6 systolic murmur heard at the third and fourth intercostal spaces along the left sternal border, points towards aortic coarctation, a congenital heart condition that could potentially explain the combination of this patient's symptoms and medical history. However, each of these complications may be potential outcomes in this patient's future. Which of these would be the most immediate and severe risk in the patient's condition? Let's dig deeper into each of the complications and understand their pathophysiology and risk factors. CLINICAL REASONING: Atrial fibrillation usually does not occur in children unless there's an associated congenital heart disease or other conditions such as hyperthyroidism. This boy's history might suggest some cardiac issues, but there's no specific finding that leads directly to fibrillation. Cor pulmonale is typically due to chronic lung disease - the only hint of lung disease in this patient is a past history of pneumonia, but this won't cause such a chronic, pervasive problem. Systemic hypertension is indeed related with coarctation of the aorta, but usually, it manifests itself as upper extremity hypertension, which wouldn't completely match this patient's presentation. Lastly, Tricuspid valve regurgitation is not a primary end-pathway or complication of aortic coarctation and hence is less likely. CONCLUDING: Considering the patient's clinical findings and their correlation with the potential complications, it seems the patient's most at-risk condition would be systemic hypertension. This is particularly due to the presence of aortic coarctation, which frequently leads to hypertension, and the presence of upper extremity hypertension in children and adolescents. RESPONSE: In light of the boy's clinical history and findings, his greatest risk would be for (C) Systemic hypertension.

**AI_D:** (1) RESTATING THE QUESTION: This scenario reports a 9-year-old boy who has not been under a physician's care for 4 years. He is on the 25th percentile for height, weight, and BMI, and has vital signs within the normal range. However, some abnormal findings include a grade 3/6 systolic murmur, weak femoral pulses juxtaposing strong brachial pulses, radiofemoral delay, signs of mild cardiomegaly and left ventricular hypertrophy from imaging. The key question is identifying which of the presented complications (Atrial fibrillation, Cor pulmonale, Systemic hypertension, or Tricuspid valve regurgitation) the boy is at the greatest risk for. (2) PLANNING: After synthesizing the information, the atypical findings guide towards a possible cardiovascular problem, specifically with the left ventricle considering the murmur location and the presence of left ventricular hypertrophy (LVH). LVH is a condition inflicted by constant forceful ejection and increased pressure within the left ventricle, causing the heart muscle to thicken over time. The weak femoral pulses alongside strong brachial pulses and radiofemoral delay might suggest the possibility of an aortic coarctation, where there is a narrowing of the lumen of the aorta, restricting blood flow from the upper to the lower body. Hypothesizing, the clinical priority might be examining the likelihood of aortic coarctation and its complications. (3) CLINICAL REASONING: Aortic coarctation is a congenital heart defect, causing a partial blocking of the aorta shortly after the arteries supplying blood to the upper body, leading to high blood pressure in the upper body and arms, in this case demonstrated by the strong brachial pulses, and lower blood pressure (leading to weak pulses) and oxygen supply in the lower parts of the body, including the legs. The grading of the murmur and the left ventricular hypertrophy could be caused by the increased afterload the left ventricle is working against because of the coarctation. (4) CONCLUDING: In this case, the long-term increased afterload on the heart over time may lead to left ventricular failure (i.e., the inability of the left ventricle to adequately pump blood to systemic circulation), which could eventually result in conditions such as systemic hypertension due to the feedback loop attempting to maintain adequate perfusion. Systemic hypertension, if left untreated, can lead to multiple complications such as stroke, heart attack, kidney damage, amongst others, and is therefore considered a major risk. (5) RESPONSE: Therefore, based on these clinical assumptions, the patient may be at greatest risk for Systemic hypertension (option C).

**AI_A:** (1) RESTATING THE QUESTION: This query seeks to determine the most likely complication a 9-year-old boy might experience given his current health status and history. He has been generally well but has a notable history of pneumonia at age 3, and recent examination unveils certain abnormal cardiovascular signs such as a systolic murmur, radiofemoral delay, mild cardiomegaly with left ventricular prominence, and left ventricular hypertrophy. The systemic effects in cardiovascular health such as intra and extra-cardiac flow, rhythm, pressure, and their valvular function need to be considered. (2) PLANNING: I'm assuming this child has a cardiac condition associated with the abnormal physical findings. It could be a congenital heart defect, or another heart disease which has resulted in the left ventricular hypertrophy and relatively late systolic murmur. One could assume that the murmur is benign. If it were, it wouldn't explain the other findings such as weak femoral pulses, radiofemoral delay, and the abnormalities seen on imaging. So the focus should be on a cardiac disease that can explain the abnormalities. A common defect that would fit the picture is coarctation of the aorta. (3) CLINICAL REASONING: Coarctation of the aorta is a congenital cardiac condition typified by narrowing of the aorta, often just distal to the left subclavian artery. This condition can cause left ventricular hypertrophy, due to increased afterload as the left ventricle works harder to pump blood through the narrowed section of the aorta. It also manifests as systolic murmurs and discrepancies in pulse strength and delay between the upper and lower body - with upper body pulses being stronger and lower body pulses being weaker and delayed, just as described in this clinical scenario. So, based on the clinical signs, it's plausible to hypothesize that this boy has coarctation of the aorta. (4) CONCLUDING: If the boy does have coarctation of the aorta, he could face several potential complications. Atrial Fibrillation is generally unlikely. Cor pulmonale, right-side heart failure due to lung disease, doesn't seem probable given the absence of lung disease signs. Tricuspid valve regurgitation refers to 'leakage' of the valve between the right atrium and right ventricle, which is also unlikely as the left side of the heart is primarily affected in coarctation of the aorta. (5) RESPONSE: However, in the context of coarctation of the aorta, systemic hypertension is plausible. Going back to the vascular physiology, you can clearly see that a downstream obstruction in a vessel like the aorta can create a high pressure upstream. This means that there'll be a high pressure burden on the vessels branching off the aorta before the site of coarctation i.e. the vessels supplying the upper body. The boy's system will try and push past the area of coarctation, raising his systemic blood pressure, particularly in the upper body. Therefore, this patient is at greatest risk for (C) Systemic hypertension."

**Facilitator** The consensus statement synthesizes the responses of AI_A, AI_B, AI_C, AI_D, and AI_E as follows: The case presents a 9-year-old boy with a significant medical history of pneumonia at age 3 and abnormal cardiovascular findings, including a systolic murmur, radiofemoral delay, mild cardiomegaly with left ventricular prominence, and left ventricular hypertrophy. These findings suggest a potential cardiac condition, possibly a congenital heart defect such as coarctation of the aorta. Coarctation of the aorta is a congenital cardiac condition characterized by a narrowing of the aorta, often just distal to the left subclavian artery. This condition can cause left ventricular hypertrophy due to increased afterload as the left ventricle works harder to pump blood through the narrowed section of the aorta. It also manifests as systolic murmurs and discrepancies in pulse strength and delay between the upper and lower body - with upper body pulses being stronger and lower body pulses being weaker and delayed, as described in this clinical scenario. Given the boy's clinical findings and their correlation with potential complications, the most immediate and severe risk in the patient's condition appears to be systemic hypertension. This is particularly due to the presence of aortic coarctation, which frequently leads to hypertension, and the presence of upper extremity hypertension in children and adolescents. Therefore, the consensus is that the patient is at greatest risk for systemic hypertension.
